# Genotype-Dependent Effect of Silencing of *TaCKX1* and *TaCKX2* on Phytohormone Crosstalk and Yield-Related Traits in Wheat

**DOI:** 10.3390/ijms222111494

**Published:** 2021-10-25

**Authors:** Bartosz Jablonski, Andrzej Bajguz, Joanna Bocian, Waclaw Orczyk, Anna Nadolska-Orczyk

**Affiliations:** 1 Department of Functional Genomics, Plant Breeding and Acclimatization Institute—National Research Institute, Radzikow, 05-870 Blonie, Poland; b.jablonski@ihar.edu.pl (B.J.); j.bocian@ihar.edu.pl (J.B.); 2 Laboratory of Plant Biochemistry, Faculty of Biology, University of Bialystok, Ciolkowskiego 1J, 15-245 Bialystok, Poland; abajguz@uwb.edu.pl; 3 Department of Genetic Engineering, Plant Breeding and Acclimatization Institute—National Research Institute, Radzikow, 05-870 Blonie, Poland; w.orczyk@ihar.edu.pl

**Keywords:** *TaCKX* expression, yield traits, wheat genotypes, awned spike, cytokinins, phytohormones

## Abstract

The influence of silenced *TaCKX1* and *TaCKX2* on coexpression of other *TaCKX* gene family members (GFMs), phytohormone regulation and yield-related traits was tested in awned-spike cultivar. We documented a strong feedback mechanism of regulation of *TaCKX* GFM expression in which silencing of *TaCKX1* upregulated expression of *TaCKX2* genes and vice versa. Additionally, downregulation of *TaCKX2* highly upregulated the expression of *TaCKX5* and *TaNAC2-5A*. In contrast, expression of these genes in silenced *TaCKX1* was downregulated. Silenced *TaCKX1* T_2_ lines with expression decreased by 47% had significantly higher thousand grain weight (TGW) and seedling root mass. Silenced *TaCKX2* T_2_ lines with expression of *TaCKX2.2.1* and *TaCKX2.2.2* decreased by 33% and 30%, respectively, had significantly higher chlorophyll content in flag leaves. *TaCKX* GFM expression, phytohormone metabolism and phenotype were additionally modified by *Agrobacterium*-mediated transformation. Two novel phytohormones, phenylacetic acid (PAA) and topolins, lack of gibberellic acid (GA) and changed phytohormone contents in the 7 days after pollination (DAP) spikes of the awned-spike cultivar compared to a previously tested, awnless one, were detected. We documented that major mechanisms of coregulation of the expression of *TaCKX* GFMs were similar in different spring wheat cultivars, but, depending on content and composition of phytohormones, regulation of yield-related traits was variously impacted.

## 1. Introduction

Wheat is globally the third most important cereal crop after maize and rice, and is mainly cultivated in moderate climates. Unfortunately, progress in yield improvement of this crop has failed to meet expectations. Study of gene function, which might play a major role in the creation of yield factors and further modelling of wheat ideotype, is one way for improvement [1].

Cytokinins (CKs) have already been presented as ‘a key driver of seed yield’ [2]. These hormones occur in plants in more than 30 forms [3] and are distributed spatially throughout the plant and temporally during all developmental stages [4]. There are two types of CKs, isoprenoid and aromatic, the first one being predominant and ubiquitous in higher plants [5,6]. The aromatics are not detected or found at low levels, e.g., 6-benzyloamino purine (BA) was found in developing wheat spikes [7,8]. Isoprenoid, free base forms, *trans*-zeatin (tZ), *cis*-zeatin (cZ), isopentenyl adenine (iP) and dihydrozeatin (DZ) as well as their ribosides (tZR, cZR, iPR, DZR) are active forms. Ribosides are considered to be the major transport forms [9,10]. The levels of CKs are controlled by biosynthesis, destruction, inactivation and translocation [5]. Long-distance translocation of root-synthesized cytokinin from xylem to phloem is essential for shoot distribution [11]. Biosynthesis is regulated by isopentenyl transferase genes (*IPT*), and destruction of tZ, cZ and iP, and their ribosides by cytokinin oxidase/dehydrogenase genes (*CKX*). Inactivation occurs via *O*- and *N*-glucosylation by cytokinin glucosyl transferases (CGTs) to storage forms. A review on *IPTs*, their function and regulation of stress and yield improvement in plants [4], and reviews on wheat *TaCKX* and *TaCGT* gene family members (GFMs) and processes in wheat have been recently reported [12,13]. Both processes of inactivation and destruction via CKX enzymes, which are especially important in the regulation of cytokinin level in cereal species, are organ- and species-specific.

The first report on regulation of grain production by *CKX* GFMs in cereals concerned rice [14], and later barley [15] and wheat [7]. Increased seed yield was also documented in other species including the model *Arabidopsis* [16] and crop oilseed rape [17]; therefore, it is suggested that cytokinins might ‘underpin the second Green revolution’ [18]. Moreover, knocking out of *HvCKX1* in barley corresponded to greater root length, hair numbers and increased surface area [19], an important component of plant morphology. Knock out mutants of *OsCKX11* in rice regulate leaf senescence and grain number by coordination of the source and sink relationship [20]. In all these cited papers, increased seed yield was affected by mutations or RNAi silencing of selected *CKX* GFMs. Positive results of modification of CK metabolism on yield parameters were also presented in natural variants of *TaCKX* GFMs. Copy number of *TaCKX4* was associated with grain weight and chlorophyll content in flag leaves [21]. Haplotype variants of *TaCKX6a02* and *TaCKX6-D1* showed a higher filling rate and greater grain size [22,23]. As reviewed, cytokinins are also important components of the response to environmental stresses [24] and mineral nutrient deficiencies [12].

According to newly revised numbering there are 13 *TaCKX* genes, basically numbered from 1 to 11, and 11 of them are allocated on each diploid genome A, B and D of allohexaploid wheat, giving a total of 35 homologues genes [12]. *TaCKX2* underwent gene duplication [25] and their paralogues were primarily subdivided into two subgene families, *TaCKX2.1* and *TaCKX2.2* [26,27]. However, according to more recent report, *TaCKX2.2* is represented by *TaCKX2.2.1*, *TaCKX2.2.2* and *TaCKX2.2.3* GFMs [12]. Silencing of *TaCKX1* or *TaCKX2.2* GFMs coordinated expression of other *CKX* GFMs, modulated cytokinins and other phytohormone contents in developing spikes and regulated yield-related traits [7,8]. A high level of silencing of *TaCKX1* strongly downregulated expression of *TaCKX11* and upregulated *TaCKX2.1*, *2.2*, *5* and *9* GFMs. This resulted in obtaining a high-yielding phenotype characterized by a higher spike number, grain number, and grain yield, but lower thousand grain weight (TGW) [7]. High silencing of *TaCKX2.2.2* was associated with strong upregulation of *TaCKX5* and *TaCKX11*, slight downregulation of *TaCKX2.2.1* but the levels of expression of other *TaCKX* GFMs were comparable to the control [8]. Silenced plants had significantly higher TGW and chlorophyll content in flag leaves, but lower grain number. These changes in coordinated expression of *TaCKX1* or *TaCKX2.2* with other GFMs resulted in diverse homeostatic balance of cytokinin forms and other phytohormones and final phenotype.

Cytokinins are suggested as signaling molecules of development [10,28]. Therefore, their crosstalk with other phytohormones, e.g., abscisic acid (ABA), gibberellins (GAs) and auxins, is well documented [29]. Both auxins and cytokinins are also necessary in processes of in vitro cereal somatic embryogenesis and plant regeneration [30]. Besides auxins, ABA is considered to have crucial importance for seed development in wheat. Higher cytokinin activity during early wheat kernel development and ABA in later stages corresponded with higher yield [31]. It was also documented in barley that crosstalk among IAA, GAs, ABA and CKs contributed to regulation of spike and spikelet development, atrophy and abortion [32].

Results of some studies on wheat spike development indicate that the level of expression of *TaCKX* GFMs and the content of phytohormones are genotypically specific. Profiles of CK forms and ABA levels varied greatly among the cultivars, and developmental kernel stages and were associated with grain yield potential of wheat cultivars [31]. Moreover, levels of expression of *TaCKX* GFMs greatly differ among the tested breeding lines and cultivars growing in a controlled environment as well as in the field [33]. Different coexpression groups of *TaCKX* GFMs, which were formed among modern varieties grown in a controlled environment and in the field, differently regulated yield-related traits [33].

In this paper new data on *TaCKX1* and *TaCKX2* silencing in the awned-spike cultivar Ostka are compared with already published data on the same GFM silencing in another awnless cultivar, Kontesa [7,8]. We found that the phenotypic result of gene silencing is predominantly dependent on the genotype of wheat. Both genotypes differ in phytohormone content and metabolism, which might influence coexpression of the silent gene with other, yield-regulating genes and consequently phytohormone homeostasis and yield-related traits. Some phytohormones, such as PAA and topolins, were newly detected and are suggested to be specific to awned-spike cultivar.

## 2. Results

### 2.1. The Levels of Silencing of TaCKX1 and TaCKX2 in T_1_

The range of relative expression of *TaCKX1* in silenced T_1_ (related to the control = 1.00) was from 0.52 to 0.74 with a mean of 0.66. The vector dedicated to silencing of *TaCKX2.2.2*-3D contained high homology of fragments of a coding sequence to silenced *TaCKX2.2.1* and *TaCKX2.1* [8]; therefore expression of all of them was measured. The range of relative expression of *TaCKX2.2.2* in silenced T_1_ was from 0.51 to 0.78 and for *TaCKX2.2.1* it was from 0.62 to 0.79. Individual T_1_ plants with the highest levels of silencing were selected.

### 2.2. Coexpression of Silenced Genes with Other TaCKX GFMs and TaNAC2-5A (NAC)

**Figure 1:**
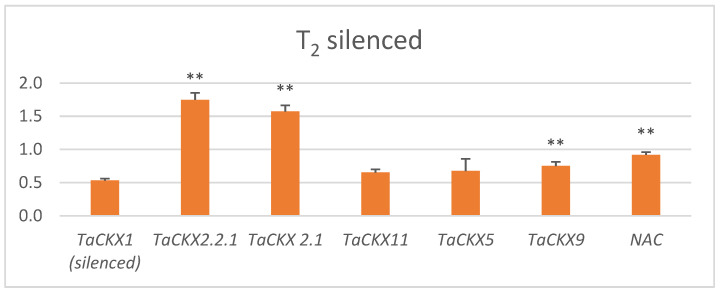
Relative values of expression of *TaCKX1* and other *TaCKX* GFMs, and *NAC* in silenced *TaCKX1* lines of T_2_. The error bars denote ± SE. Significantly different from silenced gene: ** significant at *p* ≤ 0.01; N 12 (N—number of objects/tested lines).

The range of relative expression of *TaCKX1* in selected T_2_ lines (related to the control = 1.00) was from 0.37 to 0.68 with a mean 0.53 (±0.03) (Figure 1, Table S1). These values in the group of nonsilenced plants segregated in T_2_ ranged from 0.95 to 1.42, and the mean was 1.10 (±0.05). Means of relative expression of *TaCKX11*, *TaCKX5*, *TaCKX9* and *NAC* were also decreased compared to the control, and they were 0.65 (±0.05), 0.67 (0.18), 0.75 (±0.06) and 0.92 (±0.04), respectively. These values in the case of *TaCKX11* and *TaCKX5* were close to *TaCKX1* but were significantly higher for *TaCKX9* and *NAC*. Relative values of expression of *TaCKX2.2.1*, *TaCKX2.1* were more than three times higher than in the case of the silenced gene, reaching 1.75 (±0.11) and 1.57 (±0.09), respectively.

**Figure 2:**
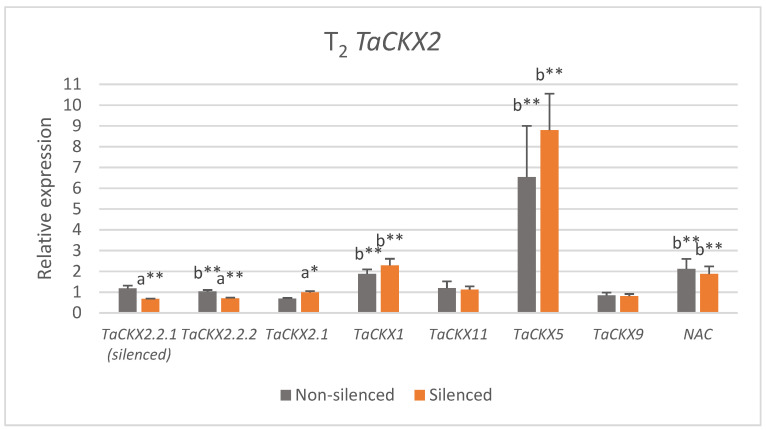
Relative values of expression of *TaCKX2* and other *TaCKX* GFMs, and *NAC* in silenced and nonsilenced *TaCKX2.2.1* lines of T_2_. The error bars denote ± SE. Different letters mean significant differences: a—between silenced and nonsilenced lines; b—between silenced *TaCKX2.2.1* and others. * significant at *p* ≤ 0.05; ** significant at *p* ≤ 0.01.

The most silenced in the group of *TaCKX2*-silenced plants was *TaCKX2.2.1*, reaching 0.67 (±0.02) (Figure 2, Table S2). In the case of nonsilenced lines, the range of expression for this gene was from 0.87 to 1.51 and the mean was 1.18 (±0.14). This value was significantly higher compared with silenced lines. A decreased level of expression compared with the control in the group of silenced plants was also observed for *TaCKX2.2.2* (0.70 ± 0.04) and *TaCKX9* (0.81 ± 0.10). Their values of expression in the group of nonsilenced plants were closer to the control, reaching 1.03 (0.07) and 0.85 (±0.13), respectively. The same values for *TaCKX2.1* in both groups were close to the control in silenced lines (0.99 ± 0.06) and lower in nonsilenced lines (0.69 ± 0.03), for *TaCKX11* slightly higher than the control (1.12 ± 0.15; 1.19 ± 0.33). In the case of *TaCKX1* and *NAC*, values of relative expression in both groups were about two times higher than silenced *TaCKX2.2.1*, reaching 2.29 (±0.32), 1.88 (±0.22) and 1.87 (±0.37), 2.12 (±0.48), respectively. These values differ significantly from the relative expression of silenced *TaCKX2.2.1*. The level of relative expression of *TaCKX5* in the group of silenced plants was more than 13 times higher (8.80 ± 1.75) and almost 10 times higher (6.53 ± 2.47) than in the most-silenced *TaCKX2.2.1*. There were significant differences in expression of *TaCKX2.2.1*, *TaCKX2.2.2* and *TaCKX2.1* between nonsilenced and silenced lines. Additionally, the relative value of expression of silenced *TaCKX2.2.1* significantly differed from nonsilenced *TaCKX2.2.2*, and nonsilenced and silenced *TaCKX1*, *TaCKX5*, and *NAC*.

### 2.3. Phytohormone Contents in Control, Nonsilenced and Silenced Lines

**Figure 3:**
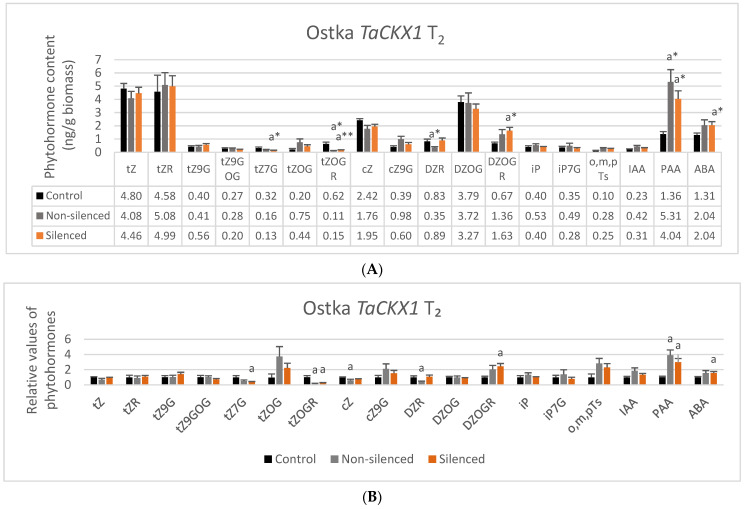
Phytohormone content (**A**) and their relative values (**B**) in control (black bars), nonsilenced (grey bars) and silenced (orange bars) *TaCKX1*, T_2_ lines. Trace amounts (<0.2 ng/g biomass): cZOGR, iPR, o,pTs, BA. Not detected: cZOG, cZR, DZ, DZ9G, DZ7G, IBA, IPA, GA. Error bars denote ± SE. Significant differences between control and nonsilenced or control and silenced lines (a): * significant at *p* ≤ 0.05; ** significant at *p* ≤ 0.01. No significant difference between nonsilenced and silenced lines.

**Figure 4:**
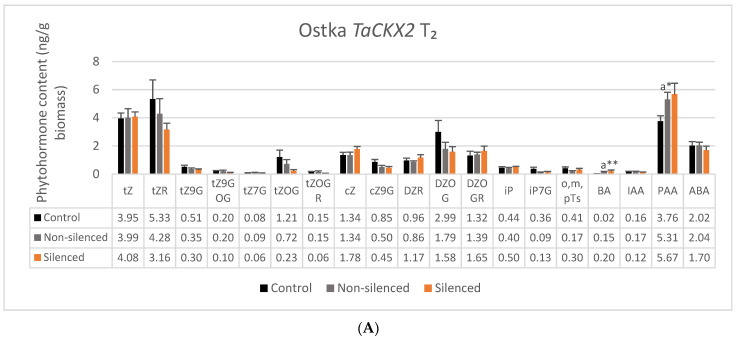

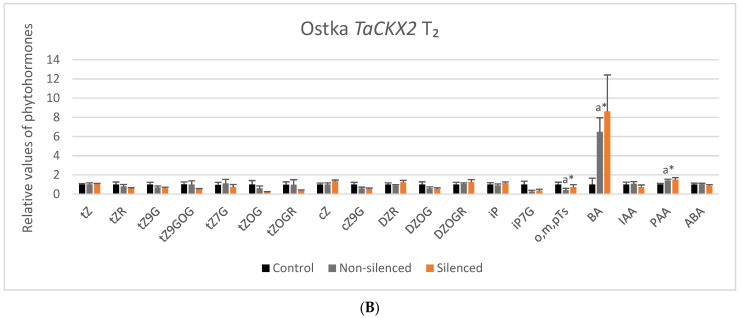
Phytohormone content (**A**) and their relative values (**B**) in control (black bars), nonsilenced (grey bars) and silenced (orange bars) *TaCKX2*, T_2_ lines. Trace amounts (<0.1 ng/g biomass): tZ7G, cZR, iPR, oT. Not detected: cZOG, cZOGR, DZ, DZ9G, DZ7G, IPA, IBA, GA. Error bars denote ± SE. Significant differences between control and nonsilenced or silenced lines (a): * significant at *p* ≤ 0.05; ** significant at *p* ≤ 0.01.

The highest contents of phytohormones measured in 7 DAP spikes of Ostka, which ranged from 1.6 to above 5 ng/g biomass, were detected for tZ, tZR, DZOG, PAA and ABA in both silenced and nonsilenced plants of *TaCKX1* and *TaCKX2* as well as in control lines (Figure 3A and Figure 4A). Contents of cZ and DZOGR were above 1 ng/g biomass, but contents of most O-glucosides and the others were below 1 ng/g biomass.

Besides significant differences in expression level of *TaCKX1* between nonsilenced and silenced plants of Ostka, there were no significant differences in contents of active, free base cytokinins, tZ, cZ, iP and other phytohormones between the same groups of lines. The only significant differences were between control and nonsilenced or control and silenced lines (Figure 3A). Already low contents of tZ7G, tZOGR and DZR were significantly lower in one or both groups of *TaCKX1*-silenced plants than in the controls. The content of DZOGR was significantly higher (more than 2 times) in silenced than in control lines. Already high content of PAA was three to four times higher in nonsilenced and silenced than in control lines. Similarly, content of ABA was significantly higher in silenced plants compared with the control. DZ and their N-glucosides were not detectable, and BA was only detected in silenced plants. Small amounts of meta-topolin (mT) and trace amounts of ortho- and para-topolins (o,pTs) are presented together as o,m,pTs.

Relative values of phytohormone content (related to the control = 1.00) are presented to show differences among values in control, nonsilenced and silenced plants of *TaCKX1* lines (Figure 3B). The values of tZOG, cZ9G, DZOGR, o,m,pTs and PAA increased two to almost four times over control in both nonsilenced and silenced lines. A smaller increase was found in the case of IAA and ABA. Relative values of tZ7G, tZOGR, cZ, DZR were significantly lower in one or both groups than in the control.

In the case of *TaCKX2* silencing, a significant difference was detected between nonsilenced and silenced plants of Ostka in expression level of *TaCKX2.2.1* and *TaCKX2.2.2*. However, there were no significant differences in contents of active, free base cytokinins, tZ, cZ, iP and other phytohormones between the same groups of lines (Figure 4A). Significant differences were only noted for BA and PAA between control and nonsilenced lines. Relative values of BA (Figure 4B) were several times higher in nonsilenced and silenced plants compared to the control. Moreover, PAA was significantly higher and o,m,pTs significantly lower in nonsilenced compared with the control lines.

### 2.4. Yield-Related Traits in Control, Nonsilenced and Silenced Lines of TaCKX1 and TaCKX2 GFMs

**Figure 5:**
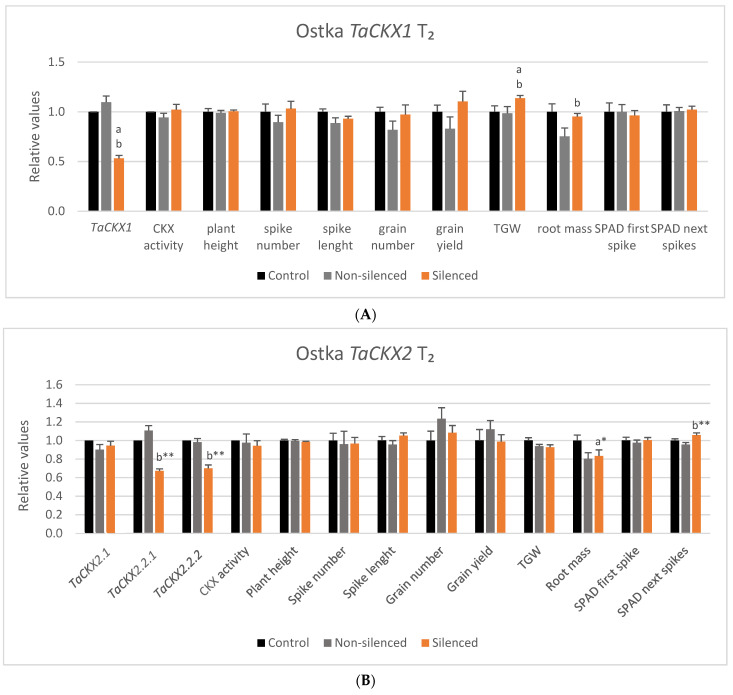
Relative values of *TaCKX1* expression and yield-related traits in *TaCKX1* (**A**) and relative values of *TaCKX2.1*, *2.2.1* and *2.2.2* expression and yield-related traits in *TaCKX2* (**B**) silenced (orange bars), nonsilenced (grey bars) and control (black bars) of T_2_ lines. Error bars denote ± SE. Significant differences between control and silenced (a) and nonsilenced and silenced (b): * significant at *p* ≤ 0.05; ** significant at *p* ≤ 0.01.

Silenced *TaCKX1* lines had significantly higher TGW than nonsilenced and control lines as well as root mass compared to nonsilenced lines (Figure 5A). Other yield-related traits were not significantly changed; however, grain yield, grain number and root mass were noticeably higher than in nonsilenced plants.

Relative expression of both *TaCKX2.2.1* and *TaCKX2.2.2* genes of silenced lines was significantly lower compared to nonsilenced ones (Figure 5B). The only significant difference between silenced and nonsilenced lines in the case of yield-related traits was higher chlorophyll content. Root mass was significantly lower in nonsilenced compared to the control. Grain number and grain yield were slightly lower in silenced compared to nonsilenced lines.

### 2.5. Coordinated Effect of TaCKX1 and TaCKX2 Silencing on Expression of Other Genes, Phytohormone Content and Yield-Related Traits

**Figure 6:**
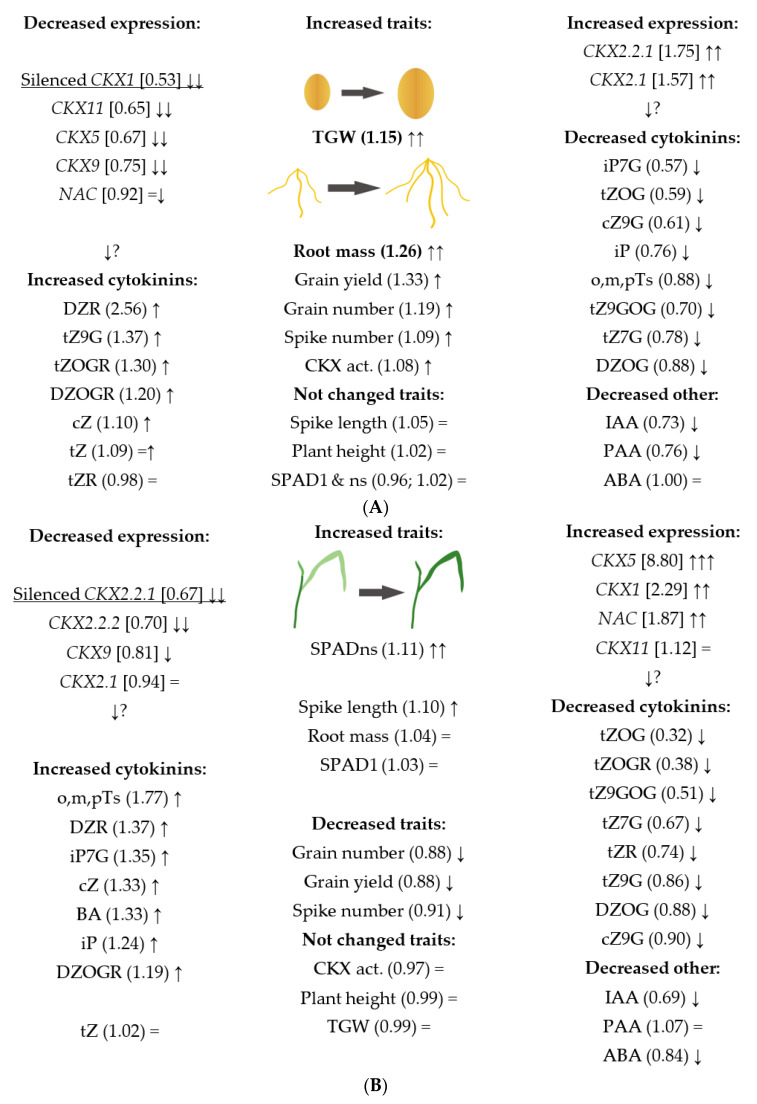
Schematic presentation of effect of *TaCKX1* (**A**) and *TaCKX2* (**B**) silencing on coregulation of expression of other *TaCKX* GFMs and phytohormone contents and yield-related traits. […] relative expression to the control; (…) RI silenced or nonsilenced; ↑↑, ↓↓ significantly increased, decreased; ↑, ↓ increased, decreased nonsignificantly.

Silenced *TaCKX1* T_2_ lines showed a 47% decreased expression (relative to the control) and 52% decreased ratio indicator (RI silenced per nonsilenced). This level of silencing was combined with significant silencing of *TaCKX11*, *TaCKX5* and *TaCKX9* and significantly increased expression of *TaCKX2.2.1* and *TaCKX2.1* (Figure 6). Silent *TaCKX1* T_2_ lines showed significantly increased TGW and root mass and increased, although not significantly, grain yield and grain number. Ratio indicators for these traits were 1.15, 1.26, 1.33 and 1.19, respectively. Other traits were not changed. The levels of phytohormones were not significantly changed. One of the most increased was DZR, with RI 2.56. The most decreased were: iP7G, tZOG, cZ9G with RI 0.57, 0.59 and 0.61, respectively. Interestingly, RI for auxins, IAA and PAA were decreased by 27% and 24% and ABA was not changed.

Silenced *TaCKX2* T_2_ lines showed significantly decreased expression for *TaCKX2.2.1* (by 33%) and expression for *TaCKX2.2.2* (by 30%); however, *TaCKX2.1* was not silenced (relative expression = 0.94). These changes of expression were related to significantly increased chlorophyll content in flag leaves of second and next spikes. RI of spike length was increased by 10%, but not significantly. Grain number, grain yield and spike number were slightly decreased. Phytohormone contents in silenced *TaCKX2* lines were not changed significantly. The highest RI, reaching 1.77, was for o,m,pTs. The most decreased cytokinins were: tZOG, tZOGR, tZ9GOG and tZ7G, with RIs of 0.32, 0.38, 0.51 and 0.67, respectively. Moreover, IAA was decreased by 31% and ABA by 16% and PAA content was similar, or slightly increased, compared to that in nonsilenced plants (1.07).

### 2.6. Correlations between Yield-Related Traits, Expression of TaCKX GFMs and NAC, and Phytohormones

There are strong differences in correlations of all tested yield-related traits with growth regulators between control and nonsilenced groups of lines (Table 1 and Table 2, Tables S3A,B and S4A,B). Although not changed in silenced *TaCKX1* lines, plant height strongly negatively correlated with decreasing content of tZOG in the control and with tZOGR in nonsilenced lines. Moreover, there was also a strong positive correlation with tZ9G and a strong negative correlation with IAA in nonsilenced lines. In contrast, the same trait in silenced lines was positively correlated with PAA. The 33% increase in grain yield and 19% increase in grain number in silenced lines (compared to nonsilenced lines) significantly positively correlated with tZ9G in silenced lines but negatively with expression of *TaCKX1* as well as DZR, IAA, and in the case of grain number with PAA in nonsilenced lines. Significantly increased in silenced lines (by 15%), TGW negatively correlated with *TaCKX2.1* in silenced lines and strongly negatively with *TaCKX1* as well as tZ7G, tZOGR, DZR and tZ in nonsilenced lines. Root mass, which is the second most significantly increased (by 26%) trait in silent *TaCKX1* lines, correlated negatively with iP in silenced lines and negatively with *TaCKX1* expression, and strongly negatively with cZ, iP7G, tZ, tZ9GOG, tZOG, and PAA in nonsilenced lines. Interestingly, CKX activity positively correlated with PAA in silenced lines and strongly positively with cZ9G in nonsilenced lines.

Nonsilenced *TaCKX1* is negatively correlated with TGW, grain number, grain yield and root mass in nonsilenced lines (Table S3). However, significantly increased expression of *TaCKX2.1* in silenced *TaCKX1* was negatively correlated with TGW. *TaCKX11* negatively correlated with spike length and positively with chlorophyll content in the 1st spike of silenced lines.

Yield-related traits in *TaCKX2.2.1*-silenced lines were correlated with other *TaCKX* GFMs and phytohormones. Plant height was negatively correlated with *TaCKX2.2.1* and positively with iP7G in silenced lines. Spike length positively correlated with *TaCKX2.2.2* and negatively with *TaCKX1* and *TaCKX9* in silenced lines, but negatively with *TaCKX2.2.1* in nonsilenced lines. This trait correlated positively with BA and ABA in silenced lines but negatively with IAA in nonsilenced lines. Grain number and grain yield correlated positively with BA in silenced but negatively with tZ7G in nonsilenced lines. There was no correlation between TGW, chlorophyll content measured by SPAD in flag leaf of 1st spike (SPAD first spike/SPAD1) and CKX activity and *TaCKX* GFMs as well as phytohormones. Chlorophyll content in flag leaves of the next spikes (SPAD next spikes/SPADns) negatively correlated with *TaCKX2.2.2* and positively correlated with DZR in silenced lines but negatively correlated with tZ7G, BA and positively correlated with cZ9G in nonsilenced lines. Root mass was negatively correlated with cZ9G in silenced lines.

### 2.7. Differences in Composition and Contents of Phytohormones in 7 DAP Spikes of Ostka vs. Kontesa

**Figure 7:**
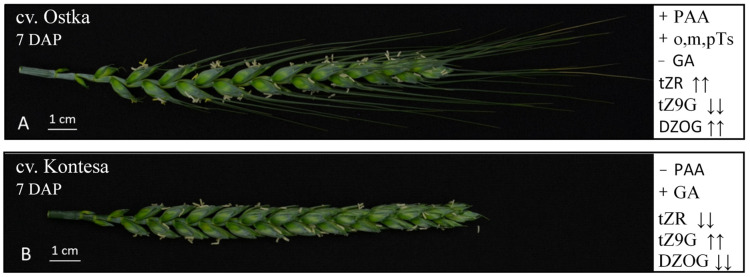
Comparison of composition and contents of phytohormones in 7 DAP spikes of Ostka and Kontesa. + newly detected; − not found; ↑↑ strongly increased; ↓↓ strongly decreased.

Two new auxins, PAA and o,m,p topolins and lack of GA were detected in 7 DAP spikes of Ostka compared to earlier data concerning 7 DAP spikes of Kontesa [7,8]. Moreover, some phytohormone contents in 7 DAP spikes of Ostka (Figure 3A and Figure 4A) and Kontesa were significantly different. These data are visualized in Figure 7, Figures S1 and S2. In *TaCKX1*-silenced plants, the content of tZR was 44 and 8 times higher in nonsilenced Ostka compared to nonsilenced Kontesa and silenced Ostka compared to silenced Kontesa, respectively. In *TaCKX2*-silenced plants, these differences were only slightly smaller and were 13 and 5 times, respectively. In contrast, the content of tZ9G in *TaCKX1*-silenced plants was 12 and 5 times lower in nonsilenced Ostka compared to nonsilenced Kontesa and silenced Ostka compared to silenced Kontesa, respectively, and in *TaCKX2*-silenced plants these differences were 5 and 10 times smaller.

Big differences between cultivars were also observed in contents of DZOG and iP. Both groups of silenced and nonsilenced lines of Ostka, independently of the silenced gene, contained about six to seven times more DZOG and two to four times more iP, although the content of the latter was below 0.5 ng/g biomass.

## 3. Discussion

The object of our research is the middle part of the 7 DAP spike of wheat. This is the early developmental stage, named the cellularization stage, which lasts up to 10 days [34], and is the middle of the cell division or cell expansion stage [35,36] or the late milk stage [31]. This stage was characterized by a high level of expression of tested *TaCKX1* and *TaCKX2* genes in wheat [27] and their orthologs in barley [37], and their silencing at this stage corresponded to yield-related traits [7,8,15,38].

### 3.1. Feedback Mechanism of Regulation of TaCKX GFM Expression and Its Coordination

Silencing of *TaCKX1* in the cultivar Ostka coordinated significantly increased expression of *TaCKX2.2.1* and *TaCKX2.1* genes, and silencing of *TaCKX2* genes caused a significant increase in expression of *TaCKX1*. A similar, but not so strong, feedback mechanism of regulation of expression was observed in the case of silencing of the same *TaCKX1* and *TaCKX2* genes in the cultivar Kontesa [7,8]. Moreover, decreased expression of *TaCKX1* in Ostka was coordinated with decreased expression of *TaCKX11*, *TaCKX5* and *TaCKX9*, but silencing of *TaCKX2* genes was coregulated with several-times increased *TaCKX5* and *NAC2*, in both silenced and nonsilenced lines. This means that regulation of expression of these two genes was mainly independent of the level of silencing of other *TaCKX* GFMs; however, it is dependent on coculture with *Agrobacterium* carrying the *TaCKX1* or *TaCKX2* silencing cassette (discussed below). This feedback mechanism of regulation of their expression resulted in stable CKX enzyme activity and maintained homeostasis of phytohormones. As reported before, most *TaCKX* GFMs are organ or tissue specific [27,33]. The *TaCKX1* and *TaCKX2* GFMs are specifically expressed in developing grains. Expression of *TaCKX5* and *TaCKX9* is specific to younger organs from seedling roots to 0 DAP spikes, but *TaCKX11* and *NAC2* are expressed in all organs [33]. Levels of relative expression of *NAC2* in roots, leaves and 14 DAP spikes reported for wheat by He et al. [39] were comparable. Moreover, orthologous to *TaCKX11* of wheat, rice *OsCKX11* [12] was, as in wheat, expressed through the plant, but predominantly in roots, leaves and panicles [20]. Coordinated decrease in silenced *TaCKX1* with *TaCKX11* was also reported by Jablonski et al. [7] and coordinated increase in *TaCKX5* with silenced *TaCKX2* GFMs was reported by Jablonski et al. [8]. Therefore, not only expression pattern, as was hypothesized before [37], but also integrated action indicate the role of *CKX* GFMs in barley and wheat development. We might also conclude that these major mechanisms of coregulation of expression of *TaCKX* GFMs are similar in different spring wheat cultivars.

### 3.2. Agrobacterium-Mediated Transformation Influences TaCKX GFM Expression, Phytohormone Metabolism and Phenotype

Different types of biotic and abiotic stress impact cytokinin homeostasis and its crosstalk with other phytohormones [24]. We might suppose that one such stress might be genetic modifications, which include in vitro culture and *Agrobacterium*-mediated transformation with different vectors. Indeed, there were significant differences in *TaCKX* GFM expression and phytohormone metabolism between in vitro origin, control F_2_ and nonsilenced T_2_ lines segregated from silenced ones. For example, in the *TaCKX1* silencing experiment, plant height was strongly negatively regulated by tZOGR and IAA and positively regulated by tZ9G in nonsilenced lines but strongly negatively regulated by tZOG in control lines. Grain number and grain yield were exclusively regulated in control lines: strongly positively by cZ and negatively by tZ and positively by DZR, respectively. Since both groups were obtained from the same in vitro culture and cultivated during the same time, under the same conditions, the only difference is that nonsilenced lines originated from in vitro culture cocultured with *Agrobacterium* carrying the respective silencing vector. Therefore, these differences might be explained by epigenetic or genetic changes in expression of plant genes influencing the process of plant transformation and their impact on the next generations.

During *Agrobacterium*-mediated transformation, transferred DNA (T-DNA) uses the host’s plant factors to be integrated to the plant genome and to express transgenes [40,41]. Regulation of T-DNA expression by DNA methylation induces the host-plant defense response by global changes in plant growth regulation. As recently reported, *Agrobacterium* VirE2, which coats single-stranded T-DNA molecules, might alter the transcriptome and proteome of the *Arabidopsis* root system to facilitate transformation [42]. Moreover, such modifications of DNA methylation and other epigenetic modifications occur during in vitro culture as well [43].

### 3.3. Phytohormonal Content and Composition Are Cultivar-Dependent

Significant changes in coregulation of expression of *TaCKX* GFMs in silenced plants do not significantly influence phytohormonal homeostasis in the tested cultivar. However, two novel phytohormones in 7 DAP spikes of the awned-spike Ostka (silenced and nonsilenced) compared to awnless Kontesa previously tested in the same conditions, were found [7,8]. The first one was the auxin PAA, detected in high concentrations, above 3 ng/g biomass. Others specifically identified in Ostka were aromatic cytokinins, and topolins with a concentration below 1 ng/g biomass. The naturally occurring auxin PAA was detected at significant levels in different tissues of higher plants, e.g., in shoots of *Triticum aestivum* [44] and young shoots of oats and barley as well as different tissues including dry seeds, inflorescences and roots of *Arabidopsis* [45]. It was indicated that this auxin activates a very similar subset of genes as IAA; therefore, both auxins might have overlapping regulatory roles. However, IAA and PAA formed slightly different auxin receptor complexes, and showed different transport characteristics, which suggested their different physiological effects [45,46]. In whole, mature grains, PAA is one of the components of phenolic acids from dietary fibers [47]. To date there is no evidence of PAA presence in developing grains, and its specialized function remains unknown.

Topolins, similarly to BA, belong to naturally occurring, aromatic CKs found in plants [48,49,50]. These CKs are products of monohydroxylation of the aromatic ring at the side chain of BA, and depending on the side there are three isomers; oT, mT and pT. Knowledge about their role in plants is lacking. However, presence of both topolins and PAA in 7 DAP spikes of Ostka testifies that they are important components of phytohormonal homeostasis in this organ.

Moreover, in contrast to Kontesa, GA was not detected in any group of silenced and nonsilenced lines of Ostka, and contents of several other phytohormones significantly differ between cultivars. The contents of tZR and DZOG were several to several dozen times higher in 7 DAP spikes of Ostka compared to Kontesa. In contrast, the content of tZ9G was several times lower in Ostka. These differences in composition and content of phytohormones indicate differences in phytohormone metabolism and transport between tested cultivars. tZR is a transporter metabolite [10], and its much higher content in 7 DAP spikes of Ostka might indicate more active transport of this riboside from roots to spikes of this cultivar. The presence of several times more DZOG in Ostka than Kontesa suggests a more active process of irreversible inactivation of DZ by O-glucosyltransferase in the former. The higher content of tZ9G and tZOG indicates higher activity of degradation of tZR by N-glucosyl transferase or O-glucosyl transferase [49] in Kontesa.

These differences in composition and content of phytohormones in 7 DAP spikes of both wheat cultivars are probably reflected in their different phenotypes. The most visible are awned spikes in Ostka and awnless spikes in Kontesa. As reported, wheat awns play an important role in photosynthesis, grain production, and drought tolerance [51,52], and awnless wheats produce more grains per spike, but their size is reduced compared to awned ones [53]. The authors suggested that allocation of assimilates to rapidly developing awns decreased spikelet number and floret fertility, and in consequence, grain number. Regulation of awn length and grain production by cytokinin metabolism was documented in rice by the *An-2* gene. The gene encodes Lonely Guy Like protein 6, which catalyzes the final step of cytokinin synthesis in *O. rufipogon* [54]. It is feasible that these new compounds of the hormonal pool in 7 DAP spikes of Ostka, PAA and topolins, are the main regulators of awns, which should be further tested.

### 3.4. Regulation of Yield-Related Traits Is Gene- and Genotype-Dependent

Silenced *TaCKX1* lines of Ostka with almost 50% decreased expression are characterized by significantly higher TGW and root mass and higher grain number and grain yield. Indeed, expression of this gene is negatively correlated with these traits in nonsilenced lines. However, decreased expression of *TaCKX1* is coexpressed with decreased expression of *TaCKX11* and increased expression of *TaCKX2.1* in both compared cultivars, Ostka and Kontesa [7]. Furthermore, TGW in silenced Ostka was significantly higher, but in Kontesa it was significantly lower compared to nonsilenced lines. Thus, silencing of *TaCKX1* coordinates expression of other *TaCKX* GFMs in different wheat cultivars in a similar way, but regulates TGW in the opposite way. Consequently, regulation of this trait is genotype-dependent. Again, higher TGW in the awned-spike cultivar Ostka is in agreement with previous discussion [53]. Regardless, other traits such as higher grain number, yield and root mass in *TaCKX1* silenced lines of both cultivars were regulated in the same manner. A similar effect of *HvCKX1* silencing on yield-related traits to Ostka and partly to Kontesa was observed in barley [15]. *TaCKX1* is an ortholog of barley *HvCKX1*, showing 90–92% homology with three homologous copies of *TaCKX1* from A, B, and D genomes. Silenced T_1_ lines of barley were characterized by higher (like in Ostka) TGW and root mass, and higher (like in both wheat cultivars) seed number and yield. It is possible that this similar mechanism of regulation of TGW between silenced *TaCKX1* lines of wheat, cultivar Ostka, and silenced *HvCKX1* lines of barley, cultivar Golden Promise, is dependent on the similar spike phenotype. While spikes of Kontesa are awnless, spikes of Ostka and Golden Promise are characterized by long awns. Activity of CKX enzyme in barley lines was decreased and correlated with higher root mass. A significant decrease in CKX enzyme activity and changed root morphology with greater root length were observed for barley lines with knockout *HvCKX1*; however, in these mutants, grain yield was not changed [19]. Based on these data we can conclude that these differences in changes of the level of CKX activity and their influence on phenotype are dependent on the number of homologous copies of *TaCKX1* and *HvCKX1* and the level or lack of gene expression. Therefore, while both genes play a very important role in yield improvement, their knock-out might not result in the expected phenotype.

In rice, the main role of *OsCKX11*, orthologous to wheat, and expressed through the plant, was coordination of the source and sink relationship by opposite regulation of leaf senescence and grain number [20]. Increased cytokinin levels in flag leaves of the Ostka mutant downregulated ABA synthesis and upregulated ABA-degrading genes, indicating antagonistic function between cytokinins and ABA. These results are difficult to compare with those of wheat, since expression of *TaCKX11* was only partly (by 35%) decreased and was strongly coordinated with decreased and increased expression of other *TaCKX* GFMs. Moreover, a noticeable increase in grain number was observed in both species.

Significantly higher chlorophyll content in flag leaves of the second and next spikes of silenced *TaCKX2* lines of Ostka is the result of 30% decreased *TaCKX2.2.2* and *TaCKX2.2.1*. Indeed, this trait was negatively regulated by *TaCKX2.2.2* expression but positively by DZ riboside, the only isoprenoid cytokinin which is not susceptible to degradation by CKX [5]. In nonsilenced plants the trait was up-regulated by cZ9G and down-regulated by tZ7G and BA. Both cZ9G and tZ7G are the products of degradation of active cZ, tZ or their ribosides by N-glucosyl-transferase, and act antagonistically in regulation of chlorophyll content. Likewise, both silenced *TaCKX2* genes act antagonistically since *TaCKX2.2.1* negatively regulates plant height and spike length and *TaCKX2.2.2* positively regulates spike length but negatively regulates chlorophyll content in flag leaves. Silencing of these genes in both wheat cultivars, Ostka and Kontesa [8], is coregulated in the same way, with increased expression of *TaCKX5* and *TaCKX1*. Additionally, silencing of *TaCKX2* in Kontesa resulted in a partly different phenotype than in Ostka, characterized by higher TGW, grain number and chlorophyll content [8]. Again, silencing of the selected *TaCKX* gene in two different spring cultivars or genotypes of wheat similarly coordinated coexpression of other *TaCKX* GFMs, but variously impacted phenotype. We documented that the effect of silencing of these two GFMs, *TaCKX1* and *TaCKX2*, gave opposite phenotypic results regarding TGW in both cultivars. Therefore, coordinated silencing of both genes, *TaCKX1* and *TaCKX2*, would increase TGW and yield more efficiently, independently of genotype.

As discussed above, leaf senescence in rice was regulated by *OsCKX11*, which antagonistically coordinated cytokinin levels and ABA [20]. However, expression of its orthologue in silenced *TaCKX2* lines of the wheat cultivar Ostka, characterized by significantly higher chlorophyll content in flag leaves, was not changed and ABA was only slightly decreased. The effect of silencing of *TaCKX2* lines of the wheat cultivar Kontesa [8] was more similar to rice and more different to Ostka. Expression of *TaCKX11* was increased, ABA content decreased by 50% and chlorophyll content in flag leaves increased. Moreover, GA level was reduced to zero and ABA level was positively correlated with *TaCKX2.2.2* expression and negatively with cZOG. Therefore, the mechanisms involved in leaf senescence of awned and awnless wheats differ.

Cytokinins and ABA are indicated as two major regulators of plant senescence; a high level of CKs might delay senescence, but high ABA content has an opposite effect [55,56]. Moreover, ABA is an important regulator of assimilate transport. Although, the content of ABA in 7 DAP spikes of both wheat cultivars was rather high (around 2 ng/g biomass); its negative correlation with chlorophyll content in flag leaves was documented only in *TaCKX2* silenced lines of Kontesa [8].

Results of regulation of yield-related traits in wheat by the *TaCKX6a02* allele selected among random, isogenic lines of wheat [22], recently renamed as *TaCKX2.1-3D* [12], were contradictory to Ostka but similar to Kontesa. Opposite data were reported for a haplotype variant of *TaCKX6-D1* [23], i.e., *TaCKX2.2.1-3D*. These contradictions suggest the importance of the level of silencing or knockout of the gene of interest in the individual genetic background. Both factors modulate other regulatory genes and take part in maintaining hormonal homeostasis.

Interestingly, higher grain number and grain yield in silenced *TaCKX1* lines of Ostka were positively correlated with tZ9G, which is the product of N-glucosyl transferase degradation of active tZ or tZR [49]. The same product was strongly positively correlated with CKX activity in nonsilenced lines. These correlations indicate a relationship between regulation of both traits by the enzyme CKX, which catalyzes degradation of tZ9G, as was observed in maize and *Arabidopsis* [49,57]. Similarly, grain yield was also significantly correlated with CKX activity and tZGs in silenced *TaCKX1* lines of Kontesa [7].

Significantly higher root mass in silenced *TaCKX1* lines of Ostka was downregulated by iP, but in nonsilenced and control lines was mainly downregulated by higher *TaCKX1* expression and cZ content. The same trait in silenced *TaCKX1* lines of Kontesa is down- or up-regulated by *TaCKX11* and *TaCKX9*, which coordinate cZOG metabolism [7]. cZOG is a product of reversible inactivation of cZ by O-glucosyl transferase [13] and was not detected in 7 DAP spikes of Ostka. However, in both cultivars, seedling root weight is upregulated mainly by cZ, which is metabolized in a different way. In contrast, root mass in silenced *TaCKX2* lines of both cultivars, Ostka and Kontesa, was not changed compared with nonsilenced lines, but in Ostka it was downregulated by cZ9G metabolism.

Unchanged plant height in silenced *TaCKX1* lines of Ostka was negatively regulated by IAA in nonsilenced and positively by PAA in silenced lines; therefore these two auxins act antagonistically. In silenced *TaCKX1* lines of Kontesa, plant height was regulated positively by GA, which is absent in 7 DAP spikes of Ostka, and positively by GA [7]. Therefore, regulation of this trait in these two cultivars is different and mainly depends on compositions and content of phytohormones.

Comparison of data of *TaCKX1* and *TaCKX2* gene silencing obtained in an awned-spike cultivar to an awnless one previously published shed new light on designing wheat ideotypes for breeding. Despite similarities of major mechanisms of coregulation of expression of tested genes with others from the same gene family in both genotypes, yield-related traits were variously impacted. We found that 7 DAP spikes of the two cultivars differ significantly in composition and content of phytohormones, which is suggested to be the main reason for the differences in the final phenotype. It was also documented that yield-related traits are modified by the process of *Agrobacterium*-mediated transformation. Moreover, it was confirmed that apart from genotype, phenotype is also dependent on the level of gene expression or silencing. In conclusion, future design of wheat ideotype for breeding should take into consideration selected genotype and requires more complex study than analysis of gene function.

## 4. Materials and Methods

### 4.1. Plant Material

Donor material was the spring wheat cultivar Ostka Smolicka provided by Plant Breeding Company Smolice Ltd., Co.—IHAR-PIB Group, Smolice 146, 63-740 Smolice, Poland. This is a high-yielding, quality (bristly) variety, class A, with high lodging resistance, high frost tolerance and awned spikes. According to our previous experiments this cultivar was susceptible to *Agrobacterium*-mediated transformation.

### 4.2. Vector Construction, Plant Transformation and Selection of Transgenic Lines

The same hpRNA type of silencing cassettes for *TaCKX1* and for *TaCKX2* silencing as previously constructed for Kontesa [7,8] were used for transformation of Ostka. Both cassettes were cloned into the binary vector pBract207 (Crop Transformation (BRACT)|John Innes Centre (jic.ac.uk), accessed on 25 October 2021) as previously reported.

Growth conditions of donor plants in a growth chamber, *Agrobacterium*-mediated transformation, and selection of putative transgenic plants were performed as we described for the cultivar Kontesa [7,8].

### 4.3. Quantitative RT-qPCR and CKX Activity Assay

Quantitative RT-qPCR was measured in 7 days after pollination (DAP) spikes of plants selected from T_1_ and T_2_ generations as previously described [7,8]. The middle part of the first spike from each experimental plant was collected, frozen in liquid nitrogen, powdered and divided for both analyses, RT-qPCR and CKX activity assay. The RT-qPCR was done for seven target genes named according to Chen et al. [12]: *TaCKX1* (JN128583), *TaCKX2.1* (JF293079), *TaCKX2.2.1* (FJ648070), *TaCKX2.2.2* (GU084177), *TaCKX11* (JN128587), *TaCKX5* and *TaCKX9* (JN128591), as well as *TaNAC2-5A* (AY625683). In brackets are accession numbers from NCBI used in previously published papers as reported by Ogonowska et al. [27]. Sequences of specific primers are shown in Supplementary Table S5. ADP-ribosylation factor (*Ref2*) (AB050957) was used as a reference gene in all qPCR reactions. The relative expression levels in transgenic plants were calculated according to expression in control, in vitro plants, set to 1.00. Transgenic plants were divided into two groups: silenced with the relative expression ≤0.79 and nonsilenced with the relative expression ≥0.8. All measurements were performed in three technical replicates in at least nine biological replicates for one group of lines. Exact numbers of plants and lines tested are included in Supplementary Materials.

CKX activity was analyzed in the second part of the sample (after grounding the sample was divided for two: one for RNA isolation and one for enzyme activity analyzes) from the same spikes as previously described [15,33].

### 4.4. Quantification of Cytokinins, Auxins, ABA and GA3

For quantification of CKs, 27 standards were used: *trans*-zeatin (tZ), *trans*-zeatin riboside (tZR), *trans*-zeatin-9-glucoside (tZ9G), *trans*-zeatin-7-glucoside (tZ7G), *trans*-zeatin-O-glucoside (tZOG), *trans*-zeatin riboside-O-glucoside (tZROG), *trans*-zeatin-9-glucoside-O-glucoside (tZ9GOG), *trans*-zeatin-9-glucoside riboside (tZ9GR), *cis*-zeatin (cZ), *cis*-zeatin-riboside (cZR), *cis*-zeatin O-glucoside (cZOG), *cis*-zeatin 9-glucoside (cZ9G), *cis*-zeatin-O-glucoside-riboside (cZROG), dihydrozeatin (DZ), dihydrozeatin-riboside (DZR), dihydrozeatin-9-glucoside (DZ9G), dihydrozeatin-7-glucoside (DZ7G), dihydrozeatin-O-glucoside (DZOG), dihydrozeatin riboside-O-glucoside (DZROG), N^6^—(Δ^2^-isopentenyl)adenine (iP), N^6^-isopentenyladenosine (iPR), N^6^-isopentenyladenosine-7-glucoside (iP7G), *para*-topolin (pT), *meta*-topolin (mT), *ortho*-topolin (oT), 6-benzylaminopurine (6-BAP). Standards for quantification of five auxins were: indole-3-acetic acid (IAA), indole-3-butyric acid (IBA), indole-3-propionic acid (IPA), 1-naphthaleneacetic acid (NAA), and 2-phenylacetic acid (PAA). Moreover, ABA and GA_3_ were quantified. The procedure is described by Jablonski et al. [7].

### 4.5. Analysis of Phenotypic Traits and Statistical Analysis

These analyses were performed as previously reported for Kontesa [8].

## Figures and Tables

**Table 1:** Regulation of yield-related traits by *TaCKX* GFMs and phytohormones in groups of silenced *TaCKX1*, nonsilenced and control plants based on correlation coefficients.

Control (N6)	Nonsilenced (N6)	Yield-Related Trait (RI Silenced/Nonsilenced)	Silenced (N12)
tZOG↓ −0.91	tZOGR↓ −0.83 **tZ9G↓ 0.85** IAA↑ −0.86 DZR↓ −0.79 DZOG↑ −0.70	Plant height (1.02) = 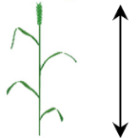	PAA↓ 0.70
iP7G↑ −0.74 DZOG↑ −0.84	*TaCKX1*↑↑ −0.78 iP7G↑ −0.89 tZ7G↑ −0.78 DZR↓ −0.72 o,m,pTs↑ −0.69	Spike length (1.05) = 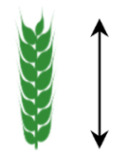	*TaCKX11*↓ −0.67
o,m,pTs↓ −0.88 np **tZ9GOG↑ 0.74**	tZ7G↑ 0.68 tZOG↑ 0.71 DZOGR↓ −0.69 **iP↑ −0.77**	Spike number (1.15) = ↑ 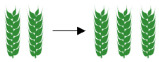	nc
**cZ↑ 0.89** tZOG↓ −0.77	*TaCKX1*↑↑ −0.66 DZR↓ −0.72 iP7G↑ −0.66 PAA↑ −0.67	Grain number (1.19) ↑ 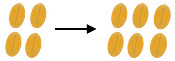	**tZ9G↑ 0.69**
**tZ↑ −0.89** np DZR↓ 0.80 np	*TaCKX1*↑↑ −0.77 tZOGR↓ −0.65 DZR↓ −0.87 iP7G↑ −0.65 IAA↑ −0.71	Grain yield (1.33) ↑ 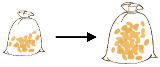	**tZ9G↑ 0.73**
o,m,pTs↓ −0.83 np **tZ↑ −0.68** **tZ9GOG↑ 0.71** **cZ9G↓ −0.70**	*TaCKX1*↑↑ −0.80 **tZ↓ −0.70** tZ7G↑ −0.86 tZOGR↓ −0.87 DZR↓ −0.77 IAA↑ −0.65	TGW (1.15) ↑↑ 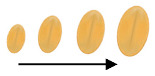	*TaCKX2.1*↑↑ −0.60
tZOG↓ 0.64 **cZ9G↓ −0.65**	nc tZ7G↑ −0.68 **cZ9G↓ 0.64**	SPAD1 (0.96) = SPADns (1.02) = 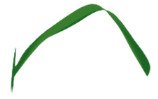	*TaCKX11*↓ 0.58
**cZ↑ −0.84** tZOG↓ 0.75 tZOGR↑↑ −0.70 o,m,pTs↓ 0.77 np	*TaCKX1*↑↑ −0.67 **cZ↓ −0.92** iP7G↑ −0.93 **tZ↓ −0.76** **tZ9GOG↑ −0.76** tZ7G↑ −0.66 tZOG↑ −0.79 o,m,pTs↑ −0.66 PAA↑ −0.72	Root mass (1.26) ↑↑ 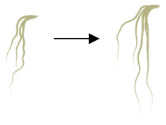	**iP↓ −0.70**
no date	**cZ9G↑ 0.93**	CKX act. (1.08) = ↑	PAA↓ 0.74

Positive (red) and negative (blue) correlation; nc—not correlated; bold—degraded by CKX; (…) ratio indicator; for N12 significant from 0.58 and for N6 significant from 0.81, (N—number of objects/tested lines); ↑, ↓—increase or decrease; ↑↑, ↓↓—significant increase or significant decrease; np—nonparametric analysis.

**Table 2:** Regulation of yield-related traits by *TaCKX* GFMs and phytohormones in groups of silenced *TaCKX2.2.1*, nonsilenced and control plants based on correlation coefficients.

Control (N9)	Nonsilenced (N10)	Yield-Related Trait (RI Silenced/Nonsilenced)	Silenced (N9) (Relative Expression of *TaCKX2.2.1* 0.51)
nc	nc	Plant height (0.99) =	*TaCKX2.2.1*↓↓ −0.88 iP7G↑ 0.80
nc	*TaCKX2.2.1* −0.67 IAA↑ −0.72	Spike length (1.10) = ↑ 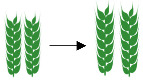	*TaCKX2.2.2*↓↓ 0.67 *TaCKX1*↑↑ −0.78 *TaCKX9*↓ −0.74 BA↑ 0.83 np ABA↓ 0.72
tZOGR↑ −0.77	**tZ↓ 0.77** np DZOG↑ 0.76 np **iP↓** 0.67 IAA↑ 0.72	Spike number (0.91) ↓ 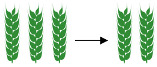	DZOG↓ 0.76 iP7G↑ 0.75 np
nc	tZ7G↑ −0.78	Grain number (0.88) ↓ 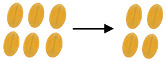	tZOGR↓ −0.67 BA↑ 0.74 np
nc	tZ7G↑ −0.78	Grain yield (0.88) ↓ 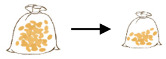	BA↑ 0.73 np
nc	nc	TGW (0.99) = 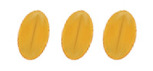	nc
	nc tZ7G↑ −0.69 np **cZ9G↑** 0.78 BA↓ −0.66	SPAD1 (1.03) = SPADns (1.11) ↑↑ 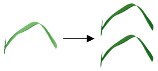	nc *TaCKX2.2.2*↓↓ −0.75 DZR↑ 0.80
DZOG↑ 0.72	nc	Root mass (1.04) = 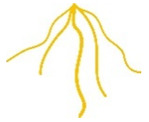	**cZ9G↓ −0.67 np**
nt	nc	CKX act. (0.97) = ↓	nc

Positive (red) and negative (blue) correlation; nc—not correlated; bold—degraded by CKX; (…) ratio indicator; for N9 significant from 0.67, (N—number of objects, or tested lines); ↑, ↓—increase or decrease; ↑↑, ↓↓—significant increase or significant decrease; np—nonparametric analysis.

## Supplementary Materials

The following are available online at https://www.mdpi.com/article/10.3390/ijms222111494/s1.
