# Supplementary material for: Genotype-Dependent Effect of Silencing of TaCKX1 and TaCKX2 on Phytohormone Crosstalk and Yield-Related Traits in Wheat
Source: Int J Mol Sci. 2021 Oct 25;22(21):11494. doi: 10.3390/ijms222111494 (PMC8584060; doi:10.3390/ijms222111494)
Supplement: Supplementary file 1 [file ijms-22-11494-s001.zip › ijms-1430054-supplementary.pdf]

Table S1. The range and the mean of relative expression in T_1 and selected T_2 lines of silenced and non-silenced *TaCKX1* lines.

	Silenced			Non-silenced		
	Min.	Mean	Max.	Min.	Mean	Max.
Ostka T_1	0.52	0.70	0.79	0.80	0.98	1.57
Ostka T_2	0.37	0.53	0.68	0.69	0.86	1.42

Table S2 A,B. The range and the mean of relative expression in T_1 and selected T_2 lines of silenced and non-silenced *TaCKX2.2.1* (A) and *TaCKX2.2.2* (B) lines.

A	Silenced			Non-silenced		
TaCKX2.2.1	Min.	Mean	Max.	Min.	Mean	Max.
Ostka T_1	0.62	0.74	0.79	0.80	1.20	2.39
Ostka T_2	0.56	0.67	0.75	0.85	1.23	1.90

B	Silenced			Non-silenced		
TaCKX2.2.2	Min.	Mean	Max.	Min.	Mean	Max.
Ostka T_1	0.51	0.68	0.78	0.80	1.09	1.53
Ostka T_2	0.42	0.69	0.79	0.81	1.13	2.35

Table S3 A,B. Correlations between yield-related traits, expression of *TaCKX* GFMs and *NAC*, and phytohormones in *TaCKX1* silenced lines (number of tested lines: N12)

A	**Spike number**	**Spike length**	**Grain number**	**Grain yield**	**TGW**	**Root mass**	**CKX activity**	***TaCKX 1***	***TaCKX 2.2.1***	***TaCKX 2.1***	***TaCKX 11***	***TaCKX 5***	***TaCKX 9***	***NAC***	**tZ**	**tZR**	**tZ9G**	**tZ9GOG**
**Plant height**	0.19	0.28	0.29	0.31	0.01	0.43	0.51	-0.01	0.42	0.00	-0.37	-0.31	0.33	-0.06	-0.35	-0.14	0.21	-0.04
**Spike number**		-0.43	**0.74**	**0.74**	-0.11	-0.17	-0.10	0.43	-0.28	0.03	0.32	0.06	0.25	0.37	-0.24	0.24	0.55	-0.18
**Spike length**			0.12	0.09	-0.07	0.01	-0.18	-0.01	0.03	-0.04	**-0.67**	-0.21	-0.36	-0.55	0.38	-0.42	0.02	-0.04
**Grain number**				**0.97**	-0.23	-0.36	-0.12	0.40	-0.28	0.11	-0.12	-0.04	0.15	-0.09	-0.22	0.20	**0.69**	0.01
**Grain yield**					0.00	-0.36	-0.04	0.38	-0.21	-0.04	-0.03	-0.18	0.21	-0.09	-0.06	0.22	**0.73**	0.01
**TGW**						0.03	0.29	-0.11	0.26	**-0.60**	0.35	-0.37	0.11	-0.07	0.07	0.11	0.15	0.08
**Root mass**							0.07	0.14	0.27	0.34	-0.24	-0.05	0.06	-0.16	-0.22	-0.20	-0.01	-0.37
**CKX activity**								-0.33	0.34	-0.16	0.04	-0.51	0.32	0.04	-0.27	-0.13	-0.06	0.40
TaCKX1									-0.08	-0.02	0.21	0.23	0.29	-0.03	-0.06	0.41	0.29	-0.36
TaCKX2.2.1										-0.53	-0.11	0.20	**0.69**	-0.22	0.01	0.10	-0.02	0.38
TaCKX2.1											-0.21	-0.33	-0.46	0.00	-0.22	-0.27	0.00	-0.22
TaCKX11												0.01	0.15	**0.70**	-0.13	0.37	-0.19	0.01
TaCKX5													0.46	0.33	-0.07	0.47	-0.27	-0.20
TaCKX9														0.07	-0.54	0.34	0.14	0.20
NAC															-0.53	0.36	-0.52	-0.33
**tZ**																-0.03	0.24	0.16
**tZR**																	-0.01	-0.20
**tZ9G**																		0.26

Gray – non-parametric analysis

B	tZ7G	tZOG	tZOGR	cZ	cZ9G	DZR	DZOG	DZOGR	iP	iP7G	o,m,pTs	BA	IAA	PAA	ABA	SPAD first spike	SPAD next spikes
**Plant height**	0.33	-0.26	-0.09	-0.43	0.12	-0.21	-0.10	0.25	-0.41	0.07	0.17	-0.27	0.14	**0.70**	0.08	-0.25	-0.18
**Spike number**	-0.16	-0.17	-0.46	-0.05	-0.22	-0.40	-0.35	0.27	0.23	-0.41	**0.64**	0.22	-0.35	-0.19	0.14	-0.16	-0.41
**Spike lenght**	-0.15	-0.04	0.30	0.07	0.13	0.23	0.13	-0.03	0.19	0.21	-0.19	0.17	0.02	0.31	-0.14	-0.38	-0.23
**Grain number**	0.02	-0.27	-0.09	0.01	0.17	-0.14	-0.07	-0.04	0.56	-0.34	0.04	0.54	-0.36	-0.18	0.16	-0.53	-0.48
**Grain yield**	0.02	-0.31	-0.13	0.01	0.05	-0.25	0.05	-0.08	0.49	-0.29	0.16	0.48	-0.21	-0.18	0.35	-0.38	-0.33
**TGW**	-0.05	0.20	0.06	0.10	0.03	-0.46	0.53	-0.13	-0.36	0.21	0.19	-0.49	0.22	-0.04	0.08	**0.67**	**0.60**
**Root mass**	0.34	-0.23	-0.12	-0.21	-0.32	-0.48	0.02	0.02	**-0.70**	-0.04	0.18	-0.22	0.52	0.27	0.15	0.31	0.11
**CKX activity**	0.35	-0.15	0.09	-0.24	0.05	-0.04	0.16	0.05	-0.52	0.31	-0.12	**-0.62**	0.42	**0.74**	0.02	0.09	0.26
TaCKX1	-0.53	0.29	**-0.76**	0.38	0.26	**-0.68**	-0.17	-0.38	0.29	0.12	0.46	0.37	-0.16	-0.35	-0.10	0.01	-0.17
TaCKX2.2.1	0.38	-0.15	0.08	-0.08	-0.27	-0.14	0.34	-0.28	-0.41	**0.62**	0.10	-0.53	0.54	0.28	0.44	-0.09	0.18
TaCKX2.1	0.30	-0.25	-0.15	-0.06	0.11	0.18	-0.18	0.04	0.10	**-0.70**	-0.25	0.27	0.14	0.00	0.02	-0.11	-0.24
TaCKX11	-0.37	**0.61**	-0.19	0.31	0.45	-0.35	-0.13	-0.03	0.06	-0.14	0.11	-0.13	-0.49	-0.25	-0.38	**0.58**	0.32
TaCKX5	-0.14	0.12	0.15	-0.14	-0.19	-0.01	-0.33	-0.04	0.21	0.34	0.15	-0.11	-0.16	-0.08	-0.01	-0.15	-0.25
TaCKX9	0.30	0.00	-0.11	-0.29	0.08	-0.37	0.11	-0.39	-0.21	0.46	0.03	-0.38	0.21	0.01	0.09	-0.07	0.25
NAC	-0.31	0.36	-0.05	-0.30	0.35	0.03	**-0.71**	0.49	-0.01	-0.25	0.09	-0.22	**-0.60**	0.33	-0.52	0.34	0.15
**tZ**	-0.52	0.16	0.23	**0.70**	-0.22	-0.03	0.47	0.16	0.34	0.18	0.14	0.16	-0.16	-0.47	0.15	-0.07	-0.20
**tZR**	-0.19	0.12	-0.26	0.15	0.05	-0.23	-0.09	-0.15	0.32	0.43	0.03	-0.21	-0.02	-0.14	0.19	-0.08	-0.10
**tZ9G**	0.22	-0.48	-0.33	0.26	-0.50	-0.44	0.48	-0.20	0.14	-0.26	0.42	0.35	0.25	-0.48	**0.72**	-0.30	-0.40
**tZ9GOG**	0.38	-0.21	0.11	0.34	-0.05	0.23	0.57	-0.29	0.00	0.19	-0.45	-0.10	0.26	-0.29	0.42	-0.36	-0.13
**tZ7G**		**-0.88**	0.20	-0.43	-0.55	0.30	0.50	-0.27	-0.38	-0.24	-0.32	-0.19	**0.73**	0.08	**0.71**	-0.16	0.22
**tZOG**			-0.27	0.40	**0.64**	-0.36	0.01	0.00	0.12	0.40	0.26	-0.18	-0.55	-0.09	**-0.78**	0.43	0.26
**tZOGR**				-0.27	-0.21	**0.82**	-0.13	0.36	0.08	-0.24	-0.48	-0.10	-0.05	0.28	0.10	-0.05	0.00
**cZ**					0.35	-0.17	0.30	-0.32	0.44	0.06	-0.18	0.30	-0.32	-0.49	-0.10	-0.04	-0.33
**cZ9G**						-0.13	-0.06	-0.13	0.33	0.10	-0.35	0.03	-0.40	0.05	**-0.60**	0.08	0.24
**DZR**							-0.17	0.25	0.34	-0.05	**-0.70**	-0.19	0.11	0.26	0.01	-0.46	-0.17
**DZOG**								**-0.65**	-0.11	0.09	-0.15	0.10	0.50	-0.43	0.45	0.12	0.32
**DZOGR**									-0.23	-0.25	0.11	-0.37	-0.54	0.51	-0.30	-0.05	-0.38
**iP**										-0.22	-0.04	0.58	-0.56	-0.35	0.01	-0.44	-0.35
**iP7G**											0.16	-0.34	0.12	0.15	-0.14	-0.07	0.20
**o,m,pTs**												-0.05	-0.25	-0.01	-0.14	0.25	-0.11
**BA**													-0.17	**-0.61**	0.12	-0.10	0.05
**IAA**														0.13	**0.62**	0.01	0.30
**PAA**															-0.25	-0.04	0.04
**ABA**																-0.33	-0.18
**SPAD first spike**																	**0.77**

Gray – non-parametric analysis

Table S4 A,B. Correlations between yield-related traits, expression of *TaCKX* GFMs and *NAC*, and phytohormones in *TaCKX2* silenced lines (number of tested lines: N9).

A	**Spike number**	**Spike length**	**Grain number**	**Grain yield**	**TGW**	**Root mass**	**CKX activity**	***TaCKX 2.1***	***TaCKX 2.2.1***	***TaCKX 2.2.2***	***TaCKX 1***	***TaCKX 11***	***TaCKX 5***	***TaCKX 9***	***NAC***	**tZ**	**tZR**	**tZ9G**
**Plant height**	0.49	0.40	**0.70**	**0.83**	0.55	-0.27	-0.01	0.21	**-0.88**	-0.10	-0.17	0.00	0.29	-0.37	0.26	-0.03	0.08	-0.11
**Spike number**		-0.35	0.17	0.23	0.27	-0.02	0.31	-0.29	-0.48	-0.32	0.58	0.44	0.60	0.46	0.64	-0.44	-0.04	-0.49
**Spike length**			0.65	**0.74**	0.38	-0.31	-0.31	0.41	-0.18	**0.67**	**-0.78**	-0.65	-0.41	**-0.74**	-0.51	0.59	0.13	0.20
**Grain number**				**0.94**	0.07	-0.32	0.01	0.40	-0.48	0.03	-0.62	-0.57	-0.34	-0.58	-0.27	0.08	0.40	-0.35
**Grain yield**					0.41	-0.41	-0.01	0.35	-0.60	0.21	-0.56	-0.47	-0.22	-0.61	-0.16	0.16	0.32	-0.19
**TGW**						-0.33	-0.03	-0.15	-0.48	0.46	0.07	0.18	0.30	-0.20	0.32	0.18	-0.15	0.31
**Root mass**							0.20	0.61	0.46	-0.39	0.21	-0.07	-0.18	0.48	0.01	0.09	-0.60	-0.08
**CKX activity**								0.14	0.16	-0.49	0.01	-0.24	-0.49	0.12	-0.19	-0.13	0.07	-0.07
TaCKX2.1									0.07	-0.11	-0.59	-0.58	-0.58	-0.40	-0.50	0.36	-0.55	0.35
TaCKX2.2.1										0.13	0.01	-0.35	-0.50	0.35	-0.39	0.20	-0.04	-0.07
TaCKX2.2.2											-0.29	-0.21	0.00	-0.32	-0.20	0.43	-0.04	0.29
TaCKX1												**0.83**	**0.78**	**0.89**	**0.79**	-0.37	-0.17	-0.27
TaCKX11													**0.93**	0.53	**0.75**	-0.35	-0.24	0.11
TaCKX5														0.50	**0.91**	-0.42	-0.13	-0.27
TaCKX9															0.54	-0.21	-0.13	-0.44
NAC																-0.64	-0.22	-0.34
**tZ**																	0.05	0.44
**tZR**																		-0.42

B	tZ9GOG	tZ7G	tZOG	tZOGR	cZ	cZ9G	DZR	DZOG	DZOGR	iP	iP7G	o,m,pTs	BA	IAA	PAA	ABA	SPAD first spike	SPAD next spikes
**Plant height**	0.11	0.18	0.60	-0.36	-0.14	-0.04	0.06	0.43	-0.31	-0.41	**0.80**	-0.12	0.40	0.50	0.27	0.40	-0.39	-0.22
**Spike number**	-0.29	0.15	-0.01	-0.25	-0.54	0.18	-0.15	**0.76**	-0.46	-0.66	**0.75**	-0.11	-0.31	0.26	-0.37	-0.17	-0.45	-0.24
**Spike lenght**	0.27	-0.20	0.18	-0.23	0.48	-0.38	-0.24	-0.13	0.11	0.33	0.16	0.02	**0.83**	-0.10	0.53	**0.72**	-0.14	-0.51
**Grain number**	-0.05	-0.05	0.08	**-0.67**	-0.01	-0.36	0.20	0.02	-0.59	-0.28	0.41	-0.05	**0.74**	0.11	0.56	0.59	-0.51	-0.20
**Grain yield**	0.15	-0.02	0.37	-0.52	0.03	-0.25	0.05	0.21	-0.12	-0.24	0.52	0.00	**0.73**	0.14	0.48	0.63	-0.53	-0.37
**TGW**	0.56	-0.20	0.32	0.26	0.03	-0.12	-0.37	0.56	0.43	-0.03	0.19	0.15	0.18	0.11	-0.15	0.19	-0.23	-0.54
**Root mass**	-0.25	0.65	-0.18	-0.35	-0.06	**-0.67**	-0.29	-0.32	0.02	0.13	-0.05	0.27	-0.60	0.07	-0.24	-0.35	0.61	0.21
**CKX activity**	0.27	0.33	0.28	-0.52	-0.21	-0.25	0.36	0.22	-0.33	-0.16	0.08	0.57	-0.29	0.02	0.09	0.00	-0.42	0.29
TaCKX2.1	0.07	0.60	0.07	**-0.72**	0.32	**-0.81**	-0.17	-0.37	-0.02	0.32	0.04	0.02	0.30	0.41	0.40	0.45	0.20	-0.08
TaCKX2.2.1	-0.04	-0.20	-0.57	0.05	0.14	-0.20	-0.14	-0.53	0.28	0.43	**-0.79**	0.47	-0.46	**-0.68**	-0.21	-0.37	0.43	0.17
TaCKX2.2.2	0.27	-0.43	-0.27	0.38	0.39	0.17	-0.61	0.03	**0.72**	0.40	-0.25	-0.08	0.31	-0.41	-0.05	0.31	-0.03	**-0.75**
TaCKX1	-0.35	0.10	-0.27	0.43	-0.40	0.33	-0.21	0.46	0.15	-0.28	0.25	-0.04	**-0.79**	-0.01	**-0.73**	**-0.72**	0.24	0.11
TaCKX11	-0.26	0.27	0.05	0.61	-0.21	0.65	-0.13	0.52	0.07	-0.21	0.30	-0.46	-0.57	0.38	-0.50	-0.42	0.16	0.05
TaCKX5	-0.35	-0.10	-0.05	0.58	-0.44	0.48	-0.29	0.48	0.10	-0.56	0.43	-0.49	-0.19	0.19	-0.59	-0.43	0.11	-0.22
TaCKX9	-0.51	0.03	-0.47	0.19	-0.31	-0.03	-0.25	0.24	0.02	-0.13	0.11	0.17	**-0.75**	-0.23	-0.66	**-0.75**	0.40	0.19
NAC	-0.08	0.02	0.20	0.38	**-0.69**	0.37	-0.20	0.37	-0.15	-0.66	0.25	-0.16	-0.64	0.12	**-0.78**	-0.65	0.02	-0.11
**tZ**	-0.07	0.08	-0.37	-0.05	**0.93**	-0.42	-0.26	0.02	0.60	**0.89**	-0.11	0.17	0.27	-0.05	0.58	0.56	0.40	-0.09
**tZR**	-0.09	**-0.77**	-0.03	0.00	0.07	0.32	0.63	0.02	-0.30	-0.04	-0.27	0.25	0.42	-0.44	0.40	0.07	-0.27	0.38
**tZ9G**	0.44	0.42	0.45	0.34	0.61	0.05	-0.11	0.04	0.57	0.62	-0.03	-0.22	0.29	0.44	0.31	0.46	0.13	-0.10
**tZ9GOG**		-0.25	0.62	0.17	-0.12	0.20	0.14	-0.13	0.10	-0.04	-0.44	0.43	0.02	-0.11	-0.05	0.07	-0.28	-0.13
**tZ7G**			0.23	-0.40	0.17	-0.28	0.00	0.22	0.00	0.08	0.55	-0.38	-0.24	**0.86**	0.17	0.25	0.10	0.25
**tZOG**				0.02	-0.12	0.20	0.57	0.00	-0.25	-0.18	0.35	0.15	0.20	0.39	0.10	0.13	-0.28	0.22
**tZOGR**					0.09	0.55	-0.13	0.06	**0.68**	0.21	-0.52	-0.19	0.04	-0.20	-0.42	-0.39	0.32	-0.02
**cZ**						-0.18	-0.12	0.01	0.63	**0.94**	-0.14	-0.10	0.38	0.10	**0.68**	0.64	0.34	-0.01
**cZ9G**							0.28	0.38	0.03	-0.23	0.19	-0.20	-0.04	0.14	-0.27	0.08	-0.45	-0.02
**DZR**								-0.22	-0.53	-0.19	0.03	0.18	0.13	0.07	0.44	-0.02	-0.22	**0.80**
**DZOG**									-0.08	-0.14	0.68	-0.19	0.09	0.34	-0.08	0.20	-0.43	-0.34
**DZOGR**										**0.70**	-0.44	0.02	-0.16	0.08	0.12	0.13	0.43	-0.20
**iP**											-0.27	0.08	0.29	-0.09	0.46	0.39	0.51	0.05
**iP7G**												-0.30	0.22	0.69	-0.08	0.33	-0.38	-0.16
**o,m,pTs**													-0.13	-0.59	-0.08	-0.30	0.12	0.28
**BA**														-0.04	**0.68**	0.66	-0.31	-0.37
**IAA**															0.30	0.41	-0.11	0.04
**PAA**																**0.82**	-0.13	0.25
**ABA**																	-0.38	-0.32
**SPAD first spike**																		0.34

Gray – non-parametric analysis

Table S5. Sequences of specific primers designed for amplification of the genes.

Gene	Name		Primer sequences	Amplicon length
Ref 2	Ta2291R	NCBI	GCTTCTGCCTGTCACATACGC	165
	Ta2291F		GCTCTCCAACAACATTGCCAAC	
TaCKX1	TaCKX1_188R		CCCAGGTACTCCTTGTACCCTAT	188
	TaCKX1_188F		GTCTACCCGCTCAACAAATCC	
TaCKX2.2.1	TaCKX2_1_R_205		TATCACATACGCCATCCATGC	205
	TaCKX2_1_F_205		TTGATCGCGGAGCTAATCCA	
TaCKX2.2.2	TaCKX2_2_R_175		ATCGTATCCTGGCCTCCTCA	175
	TaCKX2_2_F_175		TACCCCATGAACCGGAACAG	
TaCKX2.1	TaCKX2_3_R_144		TCTCCTCGTTCTGCTCCTCC	144
	TaCKX2_3_F_144		TCTACCCCATGAACCGGGAC	
TaCKX5	TaCKX5_3B_4R		CATACATGACACCAACGTACATCTT	150
	TaCKX5_3B_4F		GTCCGATTTTTGAGAAGACTGATT	
TaCKX9	TaCKX10_R_167		ACATAAAGCAATTTACCTGGACTTG	167
	TaCKX10_F_167		GAGCTAAGGGCTTGTGGGA	
TaCKX11	TaCKX3_150R		GAATTAGAGTTCACGGCTTGATG	150
	TaCKX3_150F		TTGTCAAGGGACTGTAGTAGGG	
TaNAC2-5A	TaNAC2_R		GATGATGGAGCCCAAGGCGGAG	100
	TaNAC2_F		CTGGGTGCTCTGCCGGCTCTAC	

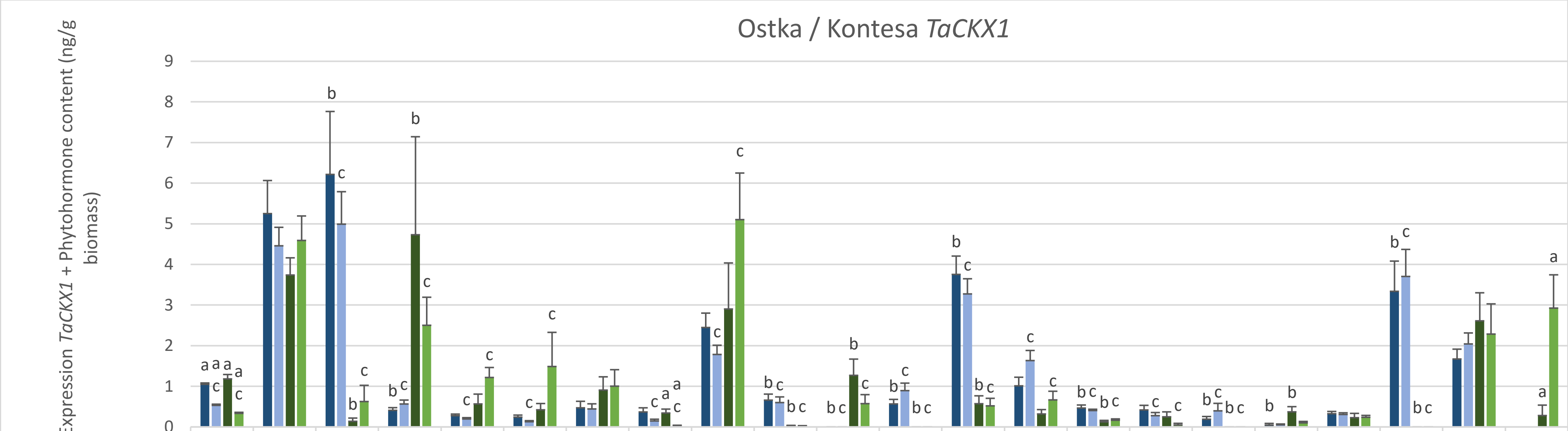

	Expr. *TaCKX1*	tZ	tZR	tZ9G	tZ9GOG	tZ7G	tZOG	tZOGR	cZ	cZ9G	cZOG	DZR	DZOG	DZOGR	iP	iP7G	oT, mT, pT	BA	IAA	PAA	ABA	GA
Ostka non-silenced	1.05	5.25	6.21	0.41	0.28	0.24	0.47	0.37	2.45	0.66	0.00	0.57	3.75	1.01	0.47	0.42	0.19	0.04	0.33	3.34	1.67	
Ostka silenced	0.53	4.46	4.99	0.56	0.20	0.13	0.44	0.15	1.78	0.60	0.00	0.89	3.27	1.63	0.40	0.28	0.40	0.05	0.31	3.70	2.04	
Kontesa non-silenced	1.18	3.74	0.14	4.73	0.57	0.42	0.91	0.34	2.90	0.03	1.27	0.00	0.57	0.32	0.12	0.25	0.00	0.37	0.23	0.00	2.61	0.28
Kontesa silenced	0.33	4.59	0.63	2.50	1.22	1.49	1.00	0.03	5.10	0.02	0.57	0.00	0.52	0.67	0.17	0.05	0.00	0.10	0.24	0.00	2.29	2.93

Figure S1. Comparison of composition and contents of phytohormones in 7 DAP spikes of non-silenced and silenced *TaCKX1* lines of Ostka vs. Kontesa. Not shown: (0.2 ng/g biomass): cZR, cZOGR, DZ, DZ9G, DZ7G, IPA, iPR. Not detected: IBA.

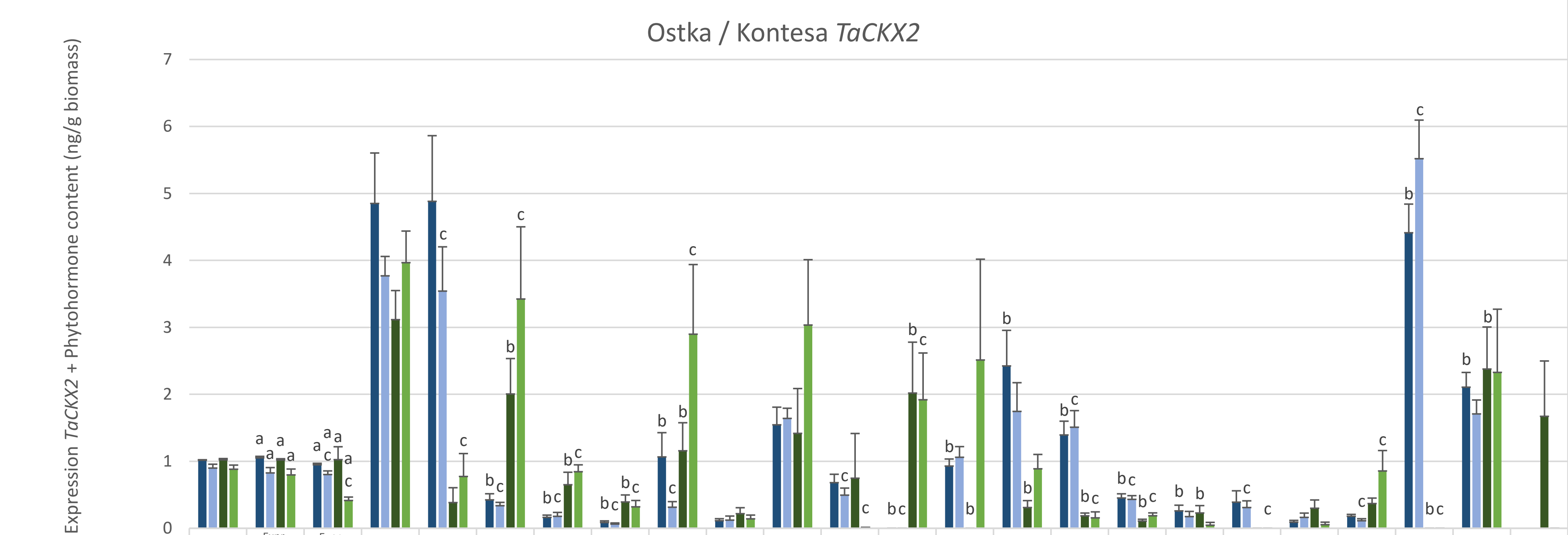

	Expr. *TaCKX2.1*	Expr. *TaCKX2.2.1*	Expr. *TaCKX 2.2.2*	tZ	tZR	tZ9G	tZ9GOG	tZ7G	tZOG	tZOGR	cZ	cZ9G	cZOG	DZR	DZOG	DZOGR	iP	iP7G	oT, mT, pT	BA	IAA	PAA	ABA	GA
Ostka non-silenced	1.02	1.05	0.95	4.85	4.88	0.42	0.16	0.09	1.07	0.11	1.55	0.68	0.00	0.93	2.42	1.39	0.45	0.26	0.39	0.09	0.18	4.41	2.11	
Ostka silenced	0.90	0.83	0.80	3.77	3.54	0.34	0.18	0.06	0.31	0.12	1.64	0.50	0.00	1.06	1.75	1.51	0.43	0.18	0.31	0.16	0.11	5.52	1.71	
Kontesa non-silenced	1.02	1.02	1.03	3.12	0.38	2.01	0.65	0.39	1.16	0.22	1.42	0.75	2.02	0.00	0.31	0.19	0.11	0.23	0.00	0.30	0.37	0.00	2.38	1.67
Kontesa silenced	0.88	0.80	0.42	3.97	0.77	3.42	0.84	0.32	2.90	0.14	3.04	0.01	1.92	2.51	0.89	0.16	0.19	0.05	0.00	0.05	0.86	0.00	2.33	0.00

Figure S2. Comparison of composition and contents of phytohormones in 7 DAP spikes of non-silenced and silenced *TaCKX2* lines of Ostka vs. Kontesa. Not shown (<0.2 ng/g biomass): DZ9G, DZ7G, cZR, cZOGR, iPR. Not detected: DZ, IBA, IPA.
